# The Siberian wood frog survives for months underwater without oxygen

**DOI:** 10.1038/s41598-018-31974-6

**Published:** 2019-01-10

**Authors:** Daniil I. Berman, Nina A. Bulakhova, Ekaterina N. Meshcheryakova

**Affiliations:** 1 Institute of Biological Problems of the North, Far East Branch, Russian Academy of Sciences, Magadan, 685000 Russia; 2 Research Institute of Biology and Biophysics, Tomsk State University, Tomsk, 634050 Russia

## Abstract

Few of the amphibian species that occur in the Subarctic and in mountains are adapted to low sub-zero temperatures; most of these species overwinter underwater. It is believed that the distribution of the species that overwinter underwater can be limited by the low oxygen levels in waterbodies covered with ice. We show that the colonisation of the coldest areas of Northern Asia (to 71°N) by the Siberian wood frog (*Rana amurensis*) was facilitated by a unique adaptation, the ability to survive extreme hypoxia — and probably anoxia — in waterbodies during overwintering. The oxygen content in the overwintering waterbodies that we have studied in different parts of the range of this species fell to 0.2–0.7 mg/L without causing any large-scale mortality among the frogs. In laboratory experiments the *R*. *amurensis* survived for up to 97 days in hermetically sealed containers with water that contained less than 0.2 mg/L oxygen at temperatures of 2–3 °C, retaining the ability to respond to external stimuli. An earlier study of a broad range of frog species has shown that very few of them can survive even brief (up to 5–7 days) exposure to oxygen-free water. The revealed adaptation to prolonged extreme hypoxia is the first known case of this kind among amphibians overwintering in water.

## Introduction

**Figure 1:**
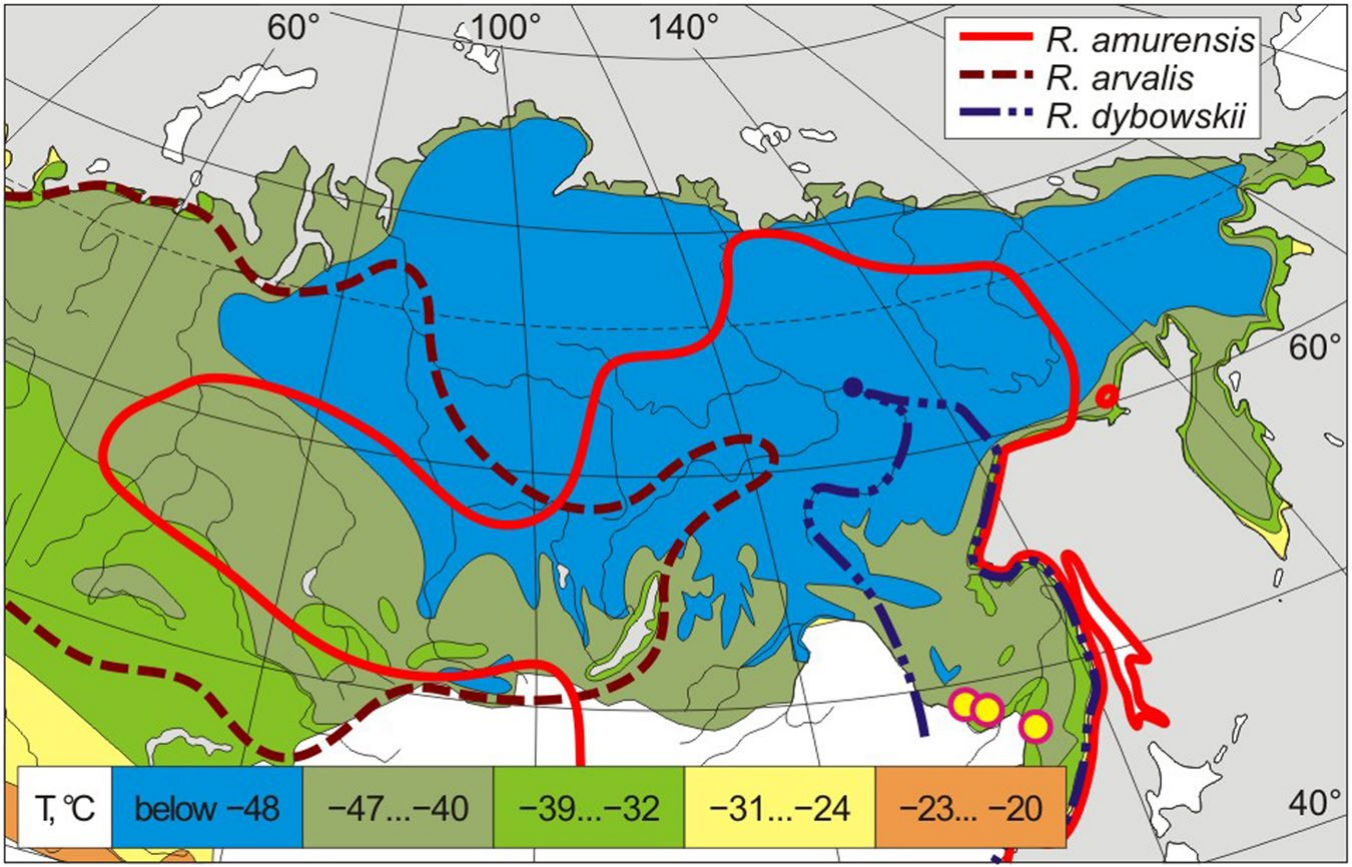
Ranges of three species of brown frogs (*Rana*) against the background of isotherms of average absolute annual minima of air temperature in Northern Asia. The boundaries of ranges are constructed by us schematically according to the list of records^9^ and our data. The isotherm circuit^29^ is modified. Yellow dots indicate the localities where the material was collected.

The adaptive strategies of northern amphibians, which allow them to survive conditions atypical of the amphibian class, have long attracted the attention of researchers^1–6^. One of the most fascinating species in this respect is the Siberian wood frog (*Rana amurensis* Boulenger, 1886), distributed in cold regions of Siberia, including the northern Subarctic and southern Arctic (to 71°N) and easternmost regions (to 154°E) (Fig. 1). Much of the range of *R*. *amurensis* is located in the permafrost zone. Judging by abundance, the optimal habitats for this species lie precisely in northeastern Eurasia, in areas with severe winters: it reaches a population density of 400–420 ind./ha in floodplains of the northern (Yakutia) and southern (Amur Oblast) parts of its range^7,8^.

It remains unclear which adaptations allowed *R*. *amurensis* to colonise the vast territory of its range, including parts of the extreme North. Since there are data indicating that *R*. *amurensis* can overwinter not only underwater but also on land, for a long time it was mistakenly assumed that this species, flourishing in areas with extremely cold winters, should be one of the most cold-tolerant among amphibians^9^. But this view was not supported by any particular studies of the cold-tolerance of this species. We have shown experimentally that the relation of *R*. *amurensis* to low temperatures is no different from that of the common frog (*R*. *temporaria* Linnaeus, 1758) or Dybowski’s frog (*R*. *dybowskii* Günther, 1876)^2,10,11^. *Rana amurensis* can survive for about 3–10 days in a supercooled state at a temperature of −2.5 °C, but in case of further cooling to lower temperatures it freezes and dies^2^. Because of this low cold resistance, it is only underwater that *R*. *amurensis* can survive the winter in cold regions. At the same time, it is known that in many Siberian waterbodies oxygen levels under ice drop almost to zero during winter, killing fish and amphibians. This extreme hypoxia takes place in lakes and rivers, both in permafrost areas and in more southern regions with a continental climate, as a result of the consumption of oxygen by the oxidation of organic compounds and functioning of photosynthesising organisms^12–15^.

Studies of threshold oxygen levels for long-term survival of frogs of the genus *Rana* in the wild are scarce, and in laboratory experiments with frogs of this genus water with 2.5–2.6 mg/L oxygen is considered severely hypoxic; few species can survive in anoxic water for a period of up to 5–7 days^16–20^. Therefore, there is an opinion that oxygen depletion is a limiting factor for the distribution of anuran amphibians^16,21,22^. At the same time, the range of *R*. *amurensis* includes large areas (e.g. the plains of Western Siberia and Central Yakutia) where oxygen is depleted in winter in most waterbodies^13,15,23,24^.

Our field studies in various parts of the range of *R*. *amurensis*, as well as some published records (see below) indicate that *R*. *amurensis* is widespread in such areas. It remained unclear whether *R*. *amurensis* is resistant to strong hypoxia or overwinters mostly in waterbodies with high levels of oxygen. To answer this question, we aimed at two goals: (1) to determine the oxygen content in the overwintering waterbodies of *R*. *amurensis* both in the north (in thermokarst lakes) and in the south (in regions without permafrost) and (2) to determine in the laboratory the threshold levels of oxygen required by *R*. *amurensis* for survival underwater.

## Results and Discussion

### Oxygen levels in overwintering waterbodies

Measurements of oxygen levels in overwintering waterbodies of *Rana amurensis* both in northern and southern parts of its range have unequivocally shown that this species does overwinter underwater at extremely low concentrations of oxygen (Table 1).

**Table 1:** Hydrological characteristics of waterbodies inhabited by *Rana amurensis* in various parts of its range.

Waterbody	Area, km^2^	Ice thickness, cm	Depth of water column under ice, cm	t, °C	Oxygen level at the bottom, mg/L	Source
**Eastern part of the range (63°N, 152°E, March)**						
“Krugloye” Lake*	0.08	80	150	2.1	0.4–0.6	Our data
“Dlinnoye” Lake	0.13	100	50	2.2	0.4–0.6	
“Podkova” quarry lake*	0.04	75	115	2–2.5	2.1	
**Southern part of the range (49°N, 130°E, March)**						
Kleshenskoye Lake*	0.25	61–76	50–104	1.1–3	0.2–0.4	Our data
Borzya River*	max width 9 m	76	61	0.5	0.3	
**Western part of the range (57°N, 84°E, January/March)**						
Meander lakes of the Ob River	max width 30 m	—	—	0.5	0.5/0.2	^30^^,^ Vorobyev S.N., Pokrovsky O.S. (personal communication), our data
Oxbow lakes*	0.04–0.19	—	—	0.4–0.7	0.6–0.9/0.5–0.7	
Large anabranch of the Ob River (estuary)	max width 50 m	—	—	0.2	1.8/1.6	
**Northern part of the range (62°N, 130°E, January/March)**						
Oxbow lakes in the environs of Yakutsk, left bank of the Lena River*	0.3–1.1	60–65/65–70	—	—	0–0.8/0–0	^14, 25^
Lakes in the Lena–Amga interfluve*	0.07–0.4	—	—	—	0.22–0.01	^8, 24^

^*^Overwintering of *R*. *amurensis* has been registered.

Dash indicates the cases in which measurements were not taken.

Thus, contrary to the opinion that winterkill caused by oxygen depletion in waterbodies that do not freeze to the bottom is the main factor limiting the distribution of *R*. *amurensis*^21,22^, this species undoubtedly overwinters underwater under hypoxic conditions.

*Rana amurensis* overwinters underwater for 6–8 months, and at least half of this period the frogs probably spend at extremely low oxygen levels (see Table 1). Mortality (from single deaths to mass mortality) of *R*. *amurensis* during the winter has been repeatedly observed by us and other authors, but it could be caused either by oxygen depletion, or freezing, or presence of hydrogen sulphide, or all above-mentioned factors together^8,21,25^.

### Survival of *Rana amurensis* under extreme hypoxic conditions

**Figure 2:**
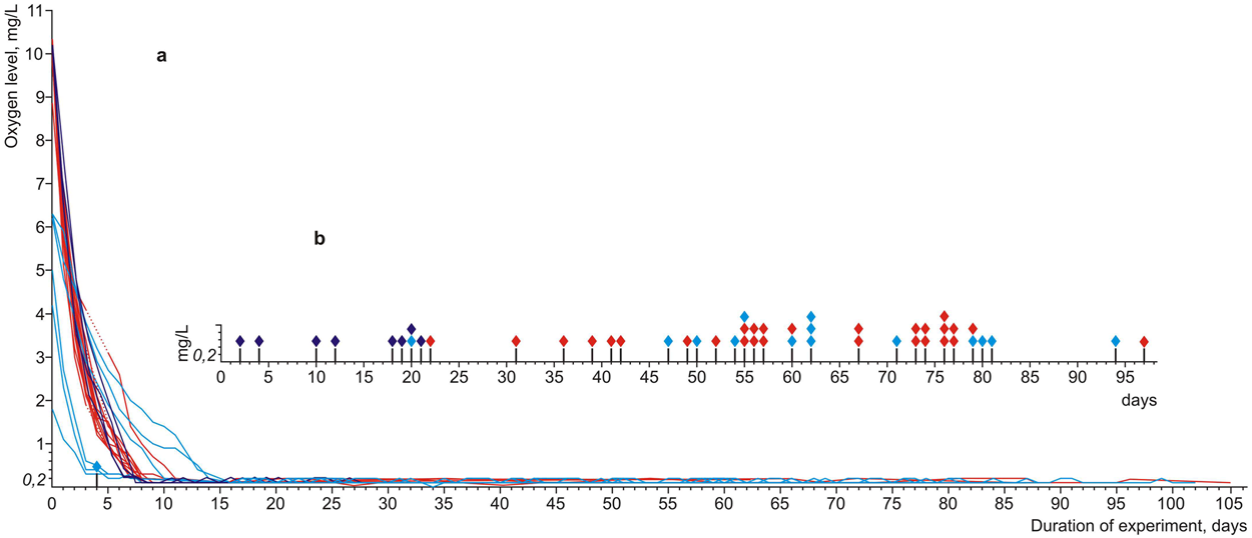
Changes in levels of dissolved oxygen in the water in three series of experiments on the capacity of *Rana amurensis* to survive hypoxia (**a**) and records of the duration of survival under extreme hypoxia conditions. (**b**) Each line refers to one hermetic container with frogs; each flag indicates the death of one individual. Experimental series I and II are marked blue and red, respectively (six containers with 15 individuals and 14 containers with 28 individuals, respectively; the frogs were captured prior to overwintering in autumn 2016 and 2017); series III is marked purple (four containers with eight individuals collected in spring 2017 after overwintering). The duration of exposure of each individual to oxygen concentrations of at most 0.2 mg/L (days) is shown on a separate scale (**b**), with flags indicating the moments of death.

Laboratory experiments were performed to determine the threshold levels of oxygen in the water that can be tolerated by *R*. *amurensis* in 20 experiments involving 2 or 3 individuals (for more details, see Supplementary Table S1). In hermetically sealed 6.3 L containers, respiration of the frogs at a temperature of 2–3 °C reduced the oxygen level in the water from the initial 1.8–10.3 mg/L to 0.2 mg/L in 6–16 days (Fig. 2a). Then the oxygen levels did not decrease further, with the dissolved oxygen meter measurements fluctuating between 0.1 and 0.2 mg/L, i.e., at the sensitivity limit of the probe^26^ (for more details see Supplementary Tables S2). The frogs, however, remained active: they swam or sat on the bottom in natural positions, to which they quickly returned after they were forcibly turned upside down. Their activity decreased only after about 14 days at an oxygen level of 0.1–0.2 mg/L: the frogs stopped swimming spontaneously, sat on the bottom, but rather slowly returned to their natural positions without visible effort after being forcibly turned upside down (i.e. remained in a state of “sluggish wakefulness”). The maximum duration of this state for individual frogs (oxygen levels of at most 0.2 mg/L and a temperature of 2–3 °C) reached 97 days; 50% individuals in the sample remained in this state longer than 60 days (Fig. 2b), retaining responses to stimuli (overturning, illumination with bright light, etc.). Only one out of 43 individuals died during the period of exposure to hypoxic conditions (one day after reaching the oxygen level of 0.3 mg/L) (Fig. 2a).

In the control group of the animals collected in autumn 2016 and 2017 and kept in open containers in water with an oxygen level no less than 5 mg/L, at temperatures of 5–7 °C or 2–3 °C, only two of the 34 individuals died, one on day 83 and one on day 90.

The dead individuals always had their hind legs outstretched, did not change their position after overturning, and did not revive after they were transferred to water with a normal oxygen level (8–10 mg/L).

The absence of further changes in the measurements of the dissolved oxygen meter after the oxygen level reached 0.1–0.2 mg/L has two alternative explanations (considering the fact that the resolution of this model of the probe is 0.1 mg/L): either oxygen was really absent in the water (i.e. the oxygen level was 0 mg/L), or the recorded oxygen level 0.1–0.2 mg/L was real, but the decrease in oxygen level was so small that the probe could not register it. It can even be suggested that if the second explanation is true, the frogs were capable of consuming extremely small amounts of oxygen, which, however, were sufficient for maintaining the vital activities of the animals.

To answer the question whether *R*. *amurensis* is capable of remaining alive for a long time by consuming oxygen at the above-mentioned extremely low concentrations, we transferred two frogs from the above-described experiment on day 54 of their exposure to water with a measured oxygen level of 0.1–0.2 mg/L from 6.3 L containers to 0.4 L containers with the same parameters of the water. The animals remained alive in the small containers for the next 24–25 days; the oxygen level, according to the probe, remained at 0.1–0.2 mg/L, which reflected the sensitivity of the probe. The water in the containers was not changed, and by the end of the experiment it was strongly polluted, probably with metabolites and small skin fragments shed by the animals (this pollution could have accelerated the mortality of the frogs). Long survival of frogs in such a small amount of water at an extremely low oxygen level probably shows that the frogs could have survived only using some other source of energy, such as anaerobic metabolism.

Moreover, frogs kept in water in autumn at an oxygen level of 5–10 mg/L and a temperature of 2–3 °C are, in a way, prepared for lack of oxygen. For example, two individuals were transferred without prior acclimation to hypoxia from the conditions described above to water with an oxygen level of 0.2 mg/L (both containers had a volume of 6.3 L). No changes could be detected in the behaviour of the animals, either during the first hours or subsequently, compared with the individuals kept under the conditions of gradually decreasing oxygen levels.

The ability to tolerate hypoxia persisted also in the frogs that left their overwintering waterbodies in spring, but to a lesser extent compared to the autumn: none of the frogs could have survived longer than 21 days at an oxygen concentration below 0.2 mg/L (Fig. 2b, Supplementary Table S3). In the control group animals collected in spring and kept in open containers in water with an oxygen level no less than 5 mg/L, at temperatures of 14–15 °C (as in natural breeding ponds) died on days 5, 8 and 15.

Thus, our experiments confirm the results of observations in the wild and show that *R*. *amurensis* clearly has a high resistance to oxygen deficiency in the water, down to 0.1–0.2 mg/L, and probably down to complete absence of oxygen. At a temperature of 2–3 °C, a frog can tolerate extreme hypoxia for up to 3 months (and possibly more), retaining the ability to respond to external stimuli. This period roughly equals half of the overwintering period of *R*. *amurensis* in the wild. It is reasonable to suggest that in this case the survival time limitation in our experiments was due to limited energy resources of the animals. This suggestion can be supported by the weak dependence (r_s_ = −0.32, p = 0.04) of the duration of survival of frogs under extreme hypoxia (days at a dissolved oxygen level lower than 0.2 mg/L; Fig. 2) on the duration of their stay (days) in the laboratory prior to the start of the experiments (see Supplementary Table S4). It is likely that some part of the individuals winters at even lower temperatures than 2–3 °C, which allows them to reduce metabolic rates and use energy resources more economical.

Tolerance of hypoxia is known in freeze tolerant amphibians^27^. It is determined by the termination of the transfer of oxygen by the blood from the organs that absorb it (lungs, mouth, pharynx or skin) to cells of the body. In our case, the hypoxia to which the frogs were exposed was determined by lack of oxygen in the water and lack of opportunity to use atmospheric oxygen.

Judging by published data, the frog species overwintering underwater that are especially tolerant to oxygen deficiency are the Common frog (*Rana temporaria*), Southern mountain yellow-legged frog (*R*. *muscosa*) and the Northern leopard frog (*R*. *pipiens*), which can survive in experiments in water at an oxygen level of 2.5–3 mg/L, as well as the tadpoles of *R*. *muscosa*, which can tolerate oxygen levels of 0.6–1.2 mg/L during overwintering in high-altitude waterbodies^16,17,28^. However, only a few frogs species (such as *R*. *pipiens*, *R*. *temporaria* and *R*. *catesbeiana*) can survive underwater without any oxygen, and only for a limited period (up to 5–7 days)^17,19^. Thus, the detected tolerance of *R*. *amurensis* to hypoxia is incomparably higher than those known in other anurans.

The described ability of *R*. *amurensis* to tolerate extreme hypoxia and probably switch to anaerobic metabolism clearly plays an important role in its geographical distribution. The ability to overwinter in oxygen-depleted waterbodies allows this species, unlike many other species of *Rana*, to inhabit southern areas of the extreme eastern Arctic, as well as the Subarctic and cold regions of Siberia, northeastern China and Mongolia.

The ability of *R*. *amurensis* to tolerate extreme hypoxia in a state of limited activity (in contrast to water turtles and similar to cyprinid fishes) for several months appears to be unique among Amphibia. It remains unclear whether adaptation to long survival of extreme hypoxia or, even more so, anoxia, is so rare among representatives of the class. Future expansion of the set of studied amphibians and their environmental conditions, especially in winter, will eventually clarify this issue.

## Methods

### Animals

*Rana amurensis* individuals (*n* = 23, weight 13.5 ± 0.9 g) were collected in the beginning of September 2016 in the environs of Arkhara village, Amur Oblast (49°N, 130°E), in April 2017 (*n* = 11, weight 21.9 ± 0.4 g), in spawning ponds near Birobidzhan, Jewish Autonomous Oblast (48°N, 132°E) and in the end of September 2017 (*n* = 54, weight 13.4 ± 0.5 g) in the environs of Lesopilnoye village, Khabarovsk Krai (46°N, 134°E). The animals were brought by plane to the Institute of the Biological Problems of the North, Far East Branch, Russian Academy of Sciences (Magadan, 59°N, 150°E) in a temperature-controlled container (5–6 °C in autumn 2016 and 14–15 °C in spring and autumn 2017) for subsequent experiments. The animals were not fed since the capture.

Frogs were collected using approved methods under appropriate permits issued by cognisant governmental agencies.

### Measurements of oxygen levels in overwintering waterbodies

Oxygen content in overwintering waterbodies of *Rana amurensis* was measured in two localities within the range of this species: in the south (49°N, 130°E, env. Arkhara village, Amur Oblast, March 2017) and in the northeast (63°N, 152°E, env. Seymchan village, Magadan Oblast, March 2015). The measurements were taken with a HACH HQ30D Flexi dissolved oxygen meter with a LDO101 luminescent sensor (accuracy 0.1 mg/L) at different depths under ice in 3–8 localities in each waterbody.

### Animal care

The conditions under which the animals captured in autumn 2016 were kept in the laboratory prior to the experiment were somewhat different from those of the others, because these animals were captured on land, whereas the others were captured in waterbodies.

The frogs collected in autumn 2016 (series I, *n* = 23) were distributed by 5–7 individuals into 1.5-L ventilated plastic containers filled with ¾ green moss, and the bottom of each container was covered with 1–1.5 cm of water. Thus, the frogs could choose to sit on the surface of the moss or stay in the water. The containers were placed in a thermostat with a temperature of 5–7 °C. During October, the transition of frogs from the surface into the overwintering waterbodies was simulated: animals in groups of 5–7 individuals were transferred from their current containers into 5-L open containers with water (temperature 5–7 °С, oxygen level 11–12 mg/L). The animals remained under these conditions for 33–67 days (Supplementary Table S4). During this period the water was not aerated, so that the oxygen level decreased to 5–8 mg/L by the beginning of the experiments. Then 15 individuals (their weight is shown in the Supplementary Table S1) were used in the experiments and 8 individuals remained in the control group. The control group of the frogs were kept in open containers in water with an oxygen level no less than 5 mg/L, at a temperature of 5–7 °C.

The frogs collected in autumn 2017 (series II, *n* = 54) were distributed by 10–12 individuals into 10-L containers filled with water (oxygen level 9–10 mg/L) and kept for 4 days at a temperature of 14–15 °C, then for 3 days at 8°С, then for 26 days at 5 °C, then at 2–3 °C, and remained under these conditions for 1–19 days (Supplementary Table S4). Then 28 individuals (their weight is shown in the Supplementary Table S1) were used in the experiments and 26 individuals remained in the control group. The control group of the frogs were kept in open containers in water with an oxygen level no less than 5 mg/L, at a temperature of 2–3 °C.

The frogs collected in spring (series III, n = 11) were kept after weighing for 7 days in 5-L open containers with water (at a temperature of 14–15 °C, as in natural breeding ponds, and oxygen level of 9–10 mg/L initially and ≥5 mg/L from day 6) prior to the beginning of the experiments. Then eight individuals (their weight is shown in the Supplementary Table S1) were used in the experiments and three individuals remained in the control group. The control group of the frogs were kept in open containers in water with an oxygen level no less than 5 mg/L, at a temperature of 14–15 °C.

All the procedures were carried out in accordance with the International Guiding Principles for Biomedical Research Involving Animals (Council for International Organizations of Medical Sciences, 1985). Rearing and experimental protocols were approved by the Bioethics Committee of Institute of Biological Problems of the North.

### Preparation of water for experiments

The water was prepared for experiments similarly in both autumn series and in the spring series. Tap water was kept in open containers for 2 days, then poured into 6.3-L containers with narrow necks (38 mm in diametre) and with hermetical screw caps and cooled to 2–3 °C. After this, the desired initial oxygen concentration in the water was achieved by pumping nitrogen gas with a nebuliser. The frogs were placed into the containers, the lids were tightened, the absence of an air bubble was checked, and the containers were placed into cooling chambers with a temperature of 2–3 °C.

### Measurement of the oxygen content in the water in the laboratory experiments

The dissolved oxygen content was measured by a HACH HQ30D Flexi digital single-channel multiparameter device with a luminescent LDO101 sensor; the accuracy of the device was 0.1 mg/L^26^. The probe was calibrated several times during the experiment at in solutions. For the experiment to determine the threshold oxygen level, the oxygen level in the water was daily measured in 2016 for the group of animals for which long survival of hypoxia was assumed. In 2017 oxygen level was measured daily until it reached 0.2 mg/L and once a week after that as well as on the day of death of animals. Measuring the dissolved oxygen in each container took at most 3–4 minutes; the water replaced by the probe was replenished with previously prepared water of the same oxygen concentration. Each measurement was repeated 4–5 times during each session. The values obtained were rounded to the tenth of a number. The tightness of the lids was regularly checked after measuring.

### Determining the threshold minimal oxygen level at which the animals can exist underwater

The water was prepared according to the protocol described in “Preparation of water for experiments”. The oxygen level in the water was set at values from 1.8 mg/L to 10.3 mg/L, two or three individuals (for more details, see Supplementary Table S1) were placed in each container, and then the containers were placed in thermostats with the same temperature. A total of 24 series of experiments were performed: 20 series in autumn and four series in spring. The volume of water displaced by immersion of the sensor into the container was replenished with specially prepared water with the same oxygen level. After the death of the last animal, the experiment with this series was terminated.

### Determining the ability of the animals to survive long in a small volume of water under extreme hypoxic conditions

It was supposed that the frogs kept in a small container would use up the minute amounts of oxygen and die, thereby demonstrating their ability to exist at extremely low oxygen levels (0.1–0.2 mg/L).

For this purpose, two individuals, after they were kept for 54 days in water with an oxygen level of at most 0.2 mg/L (6.3-L containers, water temperature 2 °C) were transferred into two transparent 0.4-L containers with the same parameters of the water and also hermetically sealed. In this experiment the containers were not opened. In this case, the oxygen level in the water was measured only before the frogs were placed into the container and at the end of the experiment. After the death of the last animal, the experiment was terminated.

### Checking the state of the animals during the experiments

The state of frogs (motor activity, level of behavioural frustration) was monitored every 24 hours by visual inspection through the transparent walls of the containers. In those experiments in which the animals were kept in 0.4-L containers, during the first 8 hours the animals were checked every 2 hours, then once a day. If the individual did not show any signs of life (movement or response to overturning), it was removed from the container, and attempts were made to revive it by placing into a container with an oxygen level of 8–10 mg/L. Each animal was used in the experiment only once.

### Statistical analysis

The dependence of survival duration at oxygen levels of at most 0.2 mg/L on the duration of keeping in the laboratory prior to the experiment was estimated using the Spearman rank correlation coefficient (r_s_).

## Electronic supplementary material

Supplementary Information

## Data Availability

All data generated or analysed during this study are included in this published article (and its Supplementary Information files).
