# Supplementary material for: The Siberian wood frog survives for months underwater without oxygen
Source: Sci Rep. 2019 Jan 10;9:13594. doi: 10.1038/s41598-018-31974-6 (PMC6365510; doi:10.1038/s41598-018-31974-6)
Supplement: Supplementary file 1 — Supplementary Information [file 41598_2018_31974_MOESM1_ESM.pdf]

Title

The Siberian wood frog survives for months underwater without oxygen

Autors

Daniil I. Berman[1], Nina A. Bulakhova[1,2]*, Ekaterina N. Meshcheryakova[1]

[1] Institute of Biological Problems of the North, Far East Branch, Russian Academy of Sciences, Magadan 685000, Russia.

[2] Research Institute of Biology and Biophysics, Tomsk State University, Tomsk 634050, Russia.

Supplementary Information

Table S1: Parameters of experiments on determining the threshold minimal oxygen level at which the *Rana amurensis* can exist underwater

Number of containers	Season (series)	Initial oxygen content, mg/L	Number of individuals, pcs.	Weight of individuals, g
1	autumn (I, II)	6.3	2	6.3, 6.7
2		6.3	2	10.1, 11.7
3		6.2	2	12.5, 14.5
4		5	3	9.0, 12.9, 16.0
5		4.1	3	13.5, 17.4, 20.1
6		1.8	3	8.0, 13.6, 22.6
7		10.1	2	12.3, 14.4
8		9.9	2	10.4, 13.7
9		9.9	2	13.2, 14.0
10		10.1	2	11.3, 11.8
11		9.8	2	12.2, 11.7
12		9.9	2	9.8, 13.3
13		10.3	2	13.9, 14.6
14		10.3	2	14.2, 14.2
15		10.3	2	10.4, 15.4
16		8.9	2	10.7, 10.7
17		10.0	2	13.0, 13.7
18		10.0	2	13.6, 14.3
19		9.8	2	10.4, 11.0
20		10.0	2	10.4, 11.2
21	spring (III)	9.6	2	21.3, 21.8
22		9.5	2	21.8, 22.6
23		9.6	2	20.1, 24.2
24		9.4	2	21.4, 22.4

Table S2: Oxygen content in water in autumn experiments and duration of survival of *Rana amurensis* under conditions to extreme hypoxia (at oxygen concentrations below 0.2 mg/L) at 2–3°C

Day of experiments	Number of containers																			
	Series I						Series II													
	1	2	3	4	5	6	7	8	9	10	11	12	13	14	15	16	17	18	19	20
1	6.3	6.3	6.2	5.0	4.2	1.8	10.1	9.9	9.9	10.1	9.8	9.9	10.3	10.3	10.3	8.9	10.0	10.0	9.8	10.0
2	5.2	5.9	4.8	2.7	2.3	1.1	6.0	6.2	6.6	6.1	6.8	5.7	6.4	6.3	5.9	5.7	5.1	5.6	6.5	6.3
3	4.6	4.7	3.9	1.6	1.2	0.8	3.5	4.2	4.4	3.8	4.4	3.8	4.1	3.6	4.1	4.2	3.2	3.0	4.6	4.6
4	3.8	3.7	2.9	0.6	0.4	**0.3**	2.1	3.0	3.4	2.1	3.0	2.7	2.4	2.2	2.8	3.0	2.2	1.9	3.2	4.1
5	3.2	2.9	2.4	0.5	0.4	0.3	1.5	1.7	2.1	1.5	1.6	1.5	1.3	1.2	1.6	1.8	–	–	–	–
6	2.7	2.4	1.9	0.3	0.2	0.3	0.9	1.2	1.4	0.9	1.0	0.9	0.9	0.9	1.3	1.5	1.0	0.9	1.5	3.1
7	2.4	1.8	1.5	0.3	0.2	0.3	0.6	0.9	1.1	0.5	0.6	0.6	0.6	0.6	0.7	0.9	0.9	0.6	1.0	2.6
8	2.0	1.5	1.1	0.2	0.2	0.2	0.5	0.6	0.7	0.1	0.3	0.3	0.2	0.3	0.2	0.7	0.4	0.4	0.5	1.4
9	1.8	1.2	0.9	0.2	0.1	0.2	0.1	0.2	0.3	0.1	0.1	0.1	–	0.2	–	0.2	0.2	0.3	0.1	1.0
10	1.5	1.0	0.5	0.1	0.1	0.1	–	–	0.3	–	–	–	–	–	–	–	–	0.1	–	0.7
11	1.4	0.9	0.2	0.2	0.2	0.2	–	–	0.2	–	–	–	–	–	–	–	–	–	–	0.5
12	1.2	0.9	0.1	0.2	0.2	0.2	–	–	–	–	–	–	–	–	–	–	–	–	–	0.2
13	0.8	0.7	0.1	0.2	0.2	0.1	0.1	0.2	0.1	0.1	0.1	0.1	0.1	0.1	0.1	0.1	0.1	0.1	0.1	0.2
14	0.4	0.5	0.1	0.2	0.2	0.1	–	–	–	–	–	–	–	–	–	–	–	–	–	–
15	0.3	0.5	0.1	0.1	0.1	0.2	–	–	–	–	–	–	–	–	–	–	–	–	–	–
16	0.2	0.2	0.1	0.1	0.1	0.2	–	–	–	–	–	–	–	–	–	–	–	–	–	–
17	0.1	0.1	0.2	0.2	0.1	0.1	–	–	–	–	–	–	–	–	–	–	–	–	–	–
18	0.1	0.1	0.2	0.1	0.2	0.1	–	–	–	–	–	–	–	–	–	–	–	–	–	–
19	0.1	0.2	0.1	0.1	0.2	0.1	–	–	–	–	–	–	–	–	–	–	–	–	–	–
20	0.1	0.1	0.1	0.1	0.1	0.1	0.1	0.1	0.1	0.1	0.1	0.04	0.1	0.04	0.04	0.1	0.1	0.1	0.1	0.1
21	0.2	0.1	0.2	0.2	0.1	0.1	–	–	–	–	–	–	–	–	–	–	–	–	–	–
22	0.2	0.2	0.2	0.1	0.2	0.1	–	–	–	–	–	–	–	–	–	–	–	–	–	–
23	0.2	0.2	0.1	0.1	0.1	0.1	–	–	–	–	–	–	–	–	–	–	–	–	–	–
24	0.2	0.2	0.2	0.2	0.2	0.1	–	–	–	–	–	–	–	–	–	–	–	–	–	–
25	0.2	0.1	0.1	0.2	0.1	0.1	–	–	–	–	–	–	–	–	–	–	–	–	–	–

26	0.1	0.2	0.1	0.1	0.1	0.1	–	–	–	–	–	–	–	–	–	–	–	–	–	–
27	0.1	0.1	0.1	0.1	0.1	0.1	0.1	0.1	0.1	0.04	0.1	0.1	0.1	0.1	0.1	0.1	0.1	0.1	0.1	0.1
28	0.1	0.1	0.1	0.1	0.1	0.2	–	–	–	–	–	–	–	–	–	–	–	–	–	–
29	0.1	0.1	0.1	0.1	0.1	0.2	–	–	–	–	–	–	–	–	–	–	–	–	–	–
30	0.1	0.1	0.1	0.1	0.1	0.2	–	–	–	–	–	–	–	–	–	–	–	–	–	–
31	0.1	0.1	0.2	0.2	0.2	0.1	–	–	–	–	–	–	–	–	–	–	–	–	–	–
32	0.1	0.1	0.1	0.1	0.1	0.1	–	–	–	–	–	–	–	–	–	–	–	–	–	–
33	0.1	0.1	0.1	0.1	0.1	0.2	–	–	**0.1**	–	–	–	–	–	–	–	–	–	–	–
34	0.1	0.1	0.1	0.1	0.1	0.1	0.1	0.1	0.1	0.2	0.1	0.1	0.1	0.1	0.1	0.1	0.1	0.1	0.1	0.1
35	0.1	0.1	0.1	0.1	0.1	0.1	–	–	–	–	–	–	–	–	–	–	–	–	–	–
36	**0.1**	0.1	0.1	0.1	0.1	0.2	–	–	–	–	–	–	–	–	–	–	–	–	–	–
37	0.2	0.2	0.1	0.1	0.1	0.1	–	–	–	–	–	–	–	–	–	–	–	–	–	–
38	0.2	0.2	0.1	0.1	0.1	0.1	–	–	–	–	–	–	–	–	–	–	–	–	–	–
39	0.2	0.2	0.1	0.1	0.1	0.1	–	–	–	–	–	–	–	–	–	–	–	–	–	–
40	0.1	0.1	0.1	0.1	0.1	0.1	**0.1**	–	–	–	–	–	–	–	–	–	–	–	–	–
41	0.1	0.1	0.1	0.2	0.2	0.1	0.1	0.1	0.1	0.1	0.1	0.1	0.1	0.1	0.1	0.1	0.1	0.1	0.1	0.1
42	0.1	0.1	0.2	0.1	0.2	0.1	–	–	–	–	–	–	–	–	–	–	–	–	–	–
43	0.2	0.2	0.2	0.1	0.1	0.1	–	–	–	–	–	–	–	–	–	–	–	–	–	–
44	0.1	0.1	0.2	0.1	0.1	0.1	–	–	–	–	–	–	–	–	–	–	–	–	–	–
45	0.1	0.1	0.2	0.1	0.1	0.1	–	–	–	–	–	–	–	–	–	–	–	–	**0.1**	–
46	0.1	0.1	0.2	0.2	0.1	0.1	–	–	–	–	–	–	–	–	–	–	–	–	–	–
47	0.1	0.1	0.1	0.1	0.1	0.1	–	–	–	–	–	–	–	–	–	–	–	–	–	–
48	0.1	0.1	0.1	0.1	0.1	0.1	0.1	0.1	0.1	0.1	0.1	**0.1**	0.1	0.1	0.1	0.1	0.1	0.1	0.1	0.1
49	0.1	0.1	0.2	0.1	0.1	0.2	–	–	–	–	–	–	–	–	–	–	–	–	–	–
50	0.2	0.1	0.1	0.1	0.1	0.2	–	–	–	–	–	–	–	**0.1**	**0.1**	–	–	–	–	–
51	0.2	0.2	0.1	0.1	0.1	0.1	–	–	–	–	–	–	–	–	–	–	–	–	–	–
52	0.1	0.2	0.1	0.1	0.1	0.2	–	–	–	–	–	–	–	–	–	–	–	–	–	–
53	0.1	0.1	0.1	0.1	**0.1**	0.2	–	–	–	–	–	–	–	–	–	–	–	–	–	–
54	0.1	0.1	0.1	0.1	0.1	0.1	–	–	–	–	–	–	–	–	–	–	–	–	–	–
55	0.1	0.1	0.1	0.1	0.1	0.1	0.1	0.1	0.1	0.1	0.1	0.1	0.1	0.1	0.2	0.1	0.1	0.1	0.1	0.2
56	0.1	0.1	0.2	0.1	0.1	0.2	–	–	–	–	–	–	–	–	–	–	–	–	–	–

57	0.1	0.1	0.2	0.1	0.1	0.2	–	–	–	–	–	–	–	–	–	–	–	–	–	–
58	0.1	0.1	0.1	0.1	0.1	0.1	–	–	–	–	–	–	–	–	–	–	**0.1**	–	–	–
59	0.1	0.1	0.2	0.2	0.2	0.2	–	–	–	–	–	–	–	–	–	–	–	–	–	–
60	0.1	0.1	0.1	0.2	0.2	0.2	–	–	–	–	–	–	–	–	–	–	–	–	–	–
61	0.2	0.2	0.1	0.1	0.2	0.1	–	–	–	–	**0.1**	–	–	–	–	–	–	–	–	–
62	0.1	0.1	0.1	0.1	0.1	0.1	0.1	0.1	0.2	0.1	0.1	0.1	0.1	0.1	0.2	0.1	0.1	0.1	0.1	0.1
63	0.1	0.1	0.2	0.1	0.2	0.1	–	–	–	**0.1**	–	–	–	–	–	–	–	–	–	–
64	0.1	0.1	0.2	0.1	0.1	0.1	–	–	–	–	–	–	–	–	–	–	–	–	–	–
65	0.1	0.1	**0.1**	0.1	0.1	0.1	–	–	–	–	–	–	–	–	–	–	**0.1**	–	**0.2**	–
66	0.2	**0.2**	0.1	0.1	0.2	0.1	–	–	–	–	–	–	–	**0.1**	–	–	*	–	*	–
67	0.1	0.1	0.2	0.1	0.1	0.1	–	–	–	–	–	–	–	*	–	–	*	–	*	**0.2**
68	0.1	0.1	0.1	0.1	0.2	0.1	–	–	–	–	–	–	–	*	**0.2**	–	*	–	*	–
69	0.1	0.1	0.1	0.1	0.1	0.1	0.2	0.1	0.1	0.1	0.1	0.1	0.1	*	*	0.1	*	0.1	*	**0.2**
70	0.1	0.1	0.1	**0.1**	0.1	**0.2**	–	–	–	–	–	–	–	*	*	–	*	–	*	*
71	0.1	**0.1**	**0.1**	0.1	0.1	0.1	–	–	–	–	–	–	–	*	*	–	*	–	*	*
72	0.1	*	*	0.1	0.1	0.2	–	–	–	–	–	–	–	*	*	–	*	–	*	*
73	0.1	*	*	0.1	0.2	0.2	–	–	–	–	–	–	–	*	*	–	*	–	*	*
74	0.1	*	*	0.1	0.2	0.1	–	–	–	–	–	–	–	*	*	–	*	–	*	*
75	0.1	*	*	0.1	0.1	0.1	–	–	–	–	–	–	–	*	*	–	*	–	*	*
76	0.1	*	*	0.1	0.2	0.1	0.1	0.1	0.1	0.1	0.1	0.1	0.1	*	*	0.2	*	–	*	*
77	0.1	*	*	0.1	**0.2**	0.2	–	–	–	–	–	–	–	*	*	–	*	**0.2**	*	*
78	**0.1**	*	*	0.1	0.1	0.1	–	–	–	–	–	–	–	*	*	–	*	*	*	*
79	*	*	*	0.1	0.1	0.2	–	–	–	–		–	–	*	*	–	*	*	*	*
80	*	*	*	0.1	0.2	0.1	–	–	–	–	–	–	–	*	*	–	*	*	*	*
81	*	*	*	0.1	0.1	0.2	–	–	–	–	–	–	–	*	*	–	*	*	*	*
82	*	*	*	0.1	0.1	0.1	–	–	–	–	**0.1**	–	**0.2**	*	*	–	*	*	*	*
83	*	*	*	0.1	0.2	0.1	0.2	0.1	–	0.1	*	0.1	*	*	*	0.1	*	*	*	*
84	*	*	*	0.1	0.1	0.1	–	–	**0.2**	–	*	–	*	*	*	–	*	*	*	*
85	*	*	*	0.1	0.1	0.1	**0.1**	–	*	–	*	**0.1**	*	*	*	**0.2**	*	*	*	*
86	*	*	*	0.1	0.1	0.1	*	**0.1**	*	–	*	*	*	*	*	–	*	*	*	*
87	*	*	*	**0.1**	**0.1**	0.1	*	*	*	–	*	*	*	*	*	–	*	*	*	*

88	*	*	*	**0.1**	*	0.2	*	*	*	–	*	*	*	*	*	**0.2**	*	*	*	*
89	*	*	*	*	*	0.1	*	*	*	–	*	*	*	*	*	*	*	*	*	*
90	*	*	*	*	*	0.1	*	*	*	0.1	*	*	*	*	*	*	*	*	*	*
91	*	*	*	*	*	0.2	*	*	*	–	*	*	*	*	*	*	*	*	*	*
92	*	*	*	*	*	0.2	*	*	*	–	*	*	*	*	*	*	*	*	*	*
93	*	*	*	*	*	0.1	*	*	*	–	*	*	*	*	*	*	*	*	*	*
94	*	*	*	*	*	0.1	*	*	*	–	*	*	*	*	*	*	*	*	*	*
95	*	*	*	*	*	0.1	*	*	*	–	*	*	*	*	*	*	*	*	*	*
96	*	*	*	*	*	0.1	*	*	*	–	*	*	*	*	*	*	*	*	*	*
97	*	*	*	*	*	0.1	*	*	*	0.2	*	*	*	*	*	*	*	*	*	*
98	*	*	*	*	*	0.1	*	*	*	–	*	*	*	*	*	*	*	*	*	*
99	*	*	*	*	*	0.1	*	*	*	–	*	*	*	*	*	*	*	*	*	*
100	*	*	*	*	*	0.2	*	*	*	–	*	*	*	*	*	*	*	*	*	*
101	*	*	*	*	*	0.1	*	*	*	–	*	*	*	*	*	*	*	*	*	*
102	*	*	*	*	*	**0.1**	*	*	*	–	*	*	*	*	*	*	*	*	*	*
103	*	*	*	*	*	*	*	*	*	–	*	*	*	*	*	*	*	*	*	*
104	*	*	*	*	*	*	*	*	*	–	*	*	*	*	*	*	*	*	*	*
105	*	*	*	*	*	*	*	*	*	**0.1**	*	*	*	*	*	*	*	*	*	*
106	*	*	*	*	*	*	*	*	*	*	*	*	*	*	*	*	*	*	*	*

Bold marked the day of the death of the individuals.
Dash indicates the cases in which measurements were not taken.
* Experiment terminated because of death of all individuals.

Table S3: Oxygen content in water in spring experiments (series III) and duration of survival of *Rana amurensis* under conditions to extreme hypoxia (at oxygen concentrations below 0.2 mg/L) at 2–3°C

Day of experiments	Number of containers			
	21	22	23	24
1	9.6	9.5	9.6	9.4
2	7.0	6.3	6.8	6.2
3	4.5	3.3	4.5	3.7
4	2.6	2.5	3.2	2.0
5	1.7	2.0	2.4	1.4
6	0.7	1.4	1.6	0.7
7	0.2	0.4	0.9	0.2
8	0.2	0.2	0.1	0.2
9	0.1	0.1	0.1	**0.1**
10	0.1	0.1	0.1	0.1
11	0.1	0.1	0.1	0.2
12	0.2	0.1	**0.2**	0.1
13	0.1	0.1	0.1	0.1
14	0.1	0.2	0.1	0.1
15	0.1	0.1	0.1	0.1
16	0.2	0.1	0.1	0.2
17	**0.1**	0.2	0.2	0.1
18	0.1	0.1	0.1	0.2
19	0.2	0.1	0.2	**0.1**
20	0.1	0.2	0.1	*
21	0.1	0.1	0.1	*
22	0.1	0.1	0.1	*
23	0.2	0.2	0.2	*
24	0.2	0.2	0.2	*
25	0.1	0.1	0.1	*
26	**0.1**	0.2	**0.1**	*
27	*	0.1	*	*
28	*	**0.1**	*	*
29	*	**0.1**	*	*
30	*	*	*	*

Bold marked the day of the death of the individuals.
* Experiment terminated because of death of all individuals.

Table S4: Duration of periods during which *Rana amurensis* were kept in the laboratory prior to of experiments (series I и II) and durations of survival under conditions to extreme hypoxia (at oxygen concentrations below 0.2 mg/L)

Total duration of keeping prior to experiments	*n*	Day of death during exposure to extreme hypoxia
33	4	31, 55, 76, 97
33	2	62, 94
40	8	22, 39, 52, 60, 73, 76, 77, 77
41	6	47, 62, 71, 79, 80, 81
44	8	41, 42, 57, 73, 74, 74, 76, 79
52	8	36, 49, 55, 56, 56, 57, 67, 67
61	2	54, 60
67	4	20, 50, 55, 62
